# AI-Assisted Chest X-Ray Interpretation in Resource-Limited Settings: LuAna Stepped-Wedge Trial Protocol

**DOI:** 10.2196/88626

**Published:** 2026-07-13

**Authors:** Maria Carolina Bueno da Silva, Paula Bresciani M de Andrade, Henrique Min Ho Lee, Pedro Vinicius Alves Silva, Ana Cristina Ferreira, Cintia Pereira Kuss, Maria Gabriela de Almeida Rodrigues, Guilherme Alberto Sousa Ribeiro, Thiago Fellipe Ortiz Camargo, William Yang Chen Fan, Pedro Vieira Santana Netto, Giovanna de Souza Mendes, Gilberto Szarf, Rafael Maffei Loureiro, Ary Serpa Neto, Joselisa Péres Queiroz de Paiva, Jaqueline Driemeyer Correia Horvath

**Affiliations:** 1 Department of Radiology, Hospital Israelita Albert Einstein, Av. Albert Einstein 627, Bldg. D, 4th Floor, 627/701, São Paulo, São Paulo, 05652-900, Brazil, 55 11998314571; 2 Department of Critical Care Medicine, Hospital Israelita Albert Einstein, São Paulo, São Paulo, Brazil; 3 Australian and New Zealand Intensive Care Research Centre (ANZIC-RC), School of Public Health and Preventive Medicine, Monash University, Melbourne, Australia; 4 Department of Intensive Care, Austin Hospital, Melbourne, Australia; 5 Department of Critical Care, Melbourne Medical School, University of Melbourne, Austin Hospital, Melbourne, Australia

**Keywords:** chest X-ray, artificial intelligence, pulmonary abnormalities, tuberculosis, cluster-randomized clinical trial, stepped wedge, protocol

## Abstract

**Background:**

Artificial intelligence (AI) has the potential to transform chest radiography interpretation by enhancing diagnostic accuracy, identifying subtle findings, reducing errors, and helping prioritize patient care. Although chest radiography remains a cost-effective and widely used imaging tool, its effectiveness is limited by overlapping anatomy and variability in clinical expertise. Integrating AI can help overcome some of these challenges, especially in resource-constrained settings. However, robust validation in real-world clinical contexts is essential before widespread implementation. This study protocol evaluates whether AI assistance improves general practitioners’ ability to detect radiographic findings on chest radiography in adults with respiratory complaints or those undergoing treatment for respiratory diseases compared with unaided interpretation. Potential benefits include increased diagnostic safety, higher physician confidence, more efficient workflows, and expanded access to expert support in underserved areas.

**Objective:**

This study aims to evaluate whether AI assistance enhances physicians’ ability to detect key radiographic abnormalities, including consolidation or pulmonary opacity, pneumothorax, atelectasis, pleural effusion, and cardiomegaly. The primary outcome is the difference in physicians’ diagnostic accuracy (per examination) when assisted by the AI tool compared with usual practice, using expert radiologist consensus as the reference value.

**Methods:**

This study is a protocol for a multicenter, stepped-wedge, cluster-randomized clinical trial following the CONSORT-AI (Consolidated Standards of Reporting Trials-Artificial Intelligence) extension and SPIRIT-AI (Standard Protocol Items: Recommendations for Interventional Trials-Artificial Intelligence) guidelines. The intervention involves the diagnostic support solution for chest radiography, Lung Analysis (LuAna), an AI-powered chest X-ray interpretation tool developed in partnership with the Brazilian Ministry of Health. Across 9 cities in Brazil, clusters will transition monthly from unaided chest X-ray interpretation by general practitioners to AI-assisted interpretation, with performance benchmarked against thoracic radiologists. The stepped-wedge design ensures that all clusters receive the intervention, reflecting real-world coordination, enhancing acceptability, improving statistical power, and strengthening causal inference through repeated measures. Diagnostic performance will be compared with a reference standard established by thoracic radiologists.

**Results:**

This project was funded in October 2024 (following ethics approval by the institutional review board). Data collection commenced in January 2026 and is projected to be completed by September 2026, marking the end of the trial period. As of November 2025, 3 centers were fully prepared for enrollment initiation. The LuAna clinical trial is currently ongoing, with data analysis (including statistical analyses) forecasted to be finalized by November 2026. Results are expected to be published by January 2027.

**Conclusions:**

This intervention is expected to enhance clinical decision-making by supporting earlier treatment initiation and more appropriate diagnostic pathways for patients with respiratory symptoms while maintaining a favorable safety profile and high physician usability. Findings from this trial will provide real-world evidence on the clinical utility of AI-assisted chest radiography. If effective, LuAna may leverage its scalability and equity advantages to become a replicable model for integrating AI into routine imaging workflows worldwide, especially in regions with limited access to specialist care.

## Introduction

Artificial intelligence (AI) is increasingly recognized as a transformative tool in global health care. In high-income countries, AI is already enhancing diagnostic accuracy, supporting clinical decision-making, accelerating research, and improving health system management [1]. However, most low- and middle-income countries (LMICs) have yet to benefit from these advances [2]. In LMICs, AI holds significant potential, enabling better care, strengthening disease surveillance, expanding telemedicine, and improving medical imaging interpretation [2]. Integrating AI in these settings could accelerate progress toward the United Nations Sustainable Development Goals [3] and promote fair access to advanced health care technologies [4].

Chest radiography is the most commonly performed imaging test worldwide and remains vital for assessing cardiothoracic conditions [5,6]. It accounts for a significant portion of the billions of radiologic examinations conducted globally each year [7]. Chest X-ray (CXR) is still a cornerstone for diagnosing pulmonary diseases, particularly where advanced imaging is unavailable. It is accessible and cost-effective [8], but its interpretation can be hindered by overlapping anatomical structures, technical limitations, and variable reader expertise [9,10]. These limitations are more pronounced in LMICs, where shortages of trained specialists contribute to diagnostic delays and errors [11].

Given its high volume and clinical relevance, CXR imaging has become a key target for developing deep-learning tools to improve diagnostic accuracy and workflow efficiency [8,12]. AI offers promising solutions by automating image analysis and supporting frontline physicians in clinical decision-making. Advances in convolutional neural networks have enabled the development of AI tools with diagnostic accuracy comparable to radiologists for key findings, such as pneumonic consolidation, pneumothorax, and pulmonary nodules [4,6,13]. A recent study shows that an AI system using deep convolutional neural networks could reliably analyze chest radiographs to identify features of COVID-19 pneumonia, such as ground-glass opacities and consolidation, offering a faster, cheaper alternative to computed tomography (CT) scans [14]. Though accuracy decreased for non–COVID-19 abnormalities poorly represented in the training data, it remained useful for screening and confirming typical cases, with suspicious cases requiring a CT scan. Ongoing improvements and diverse data will boost its performance, making AI-assisted CXR evaluation practical and cost-effective. However, 2 persistent challenges remain: (1) integrating AI into real-world clinical workflows and (2) providing effective support for nonradiologist physicians in resource-limited environments.

Rigorous validation is essential before implementing AI tools. This process requires not only comparisons with expert diagnoses to establish accuracy and reliability, but also prospective evaluation in real-world clinical settings to confirm safety, usability, and cost-effectiveness. Despite being resource-intensive, such efforts have already shown tangible benefits: in lung cancer pathways, adjunct AI for CXR interpretation can reduce missed cancers and is expected to be cost-saving compared with radiologist-only reporting [15,16].

In tuberculosis (TB) control, economic models from Pakistan indicate that AI-based computer-aided CXR triage can reduce diagnostic costs by approximately 20% to 40% compared with standard smear- or GeneXpert-based algorithms, primarily by limiting unnecessary confirmatory testing while maintaining case detection [17,18]. Cost-effectiveness is strongest in moderate- to high-prevalence settings and high-throughput programs, where economies of scale reduce per-screen costs. Similarly, evidence from Zambia shows that comprehensive radiography-based case-finding interventions can improve detection and achieve favorable incremental cost-effectiveness compared with passive case detection [19]. Overall, economic value is driven by prevalence, screening volume, and test cost differentials, with added system-level benefits including earlier diagnosis and more efficient resource allocation [15-19].

Despite the promising retrospective performance of AI models for CXR interpretation, their real-world effectiveness remains uncertain when deployed across large, heterogeneous health systems. Models trained and validated in limited or single-center datasets frequently experience distribution shifts that degrade performance when applied to different populations, imaging equipment, and clinical workflows [20]. A continental-scale public health system, such as Brazil’s Unified Health System (Sistema Único de Saúde [SUS]), encompasses substantial geographic, demographic, and operational heterogeneity, making it an ideal yet challenging environment to evaluate clinical utility and equity of AI tools. Therefore, prospective, multicenter implementation trials that measure the impact of AI on clinician performance and patient management across diverse, real-world settings are essential to establish generalizability, robustness, and fairness prior to widescale adoption [20,21].

The LuAna (Lung Analysis) trial will evaluate whether AI-assisted CXR interpretation improves the detection of radiographic findings in adults with respiratory symptoms or undergoing treatment for respiratory diseases compared with physician-only interpretation within Brazil’s Unified Health System. We hypothesize that AI assistance will enhance diagnostic accuracy, expedite triage, reduce delays, improve cost-effectiveness, and facilitate scalability. If successful, LuAna could become a model for equitable AI integration into diagnostic workflows in LMICs and beyond.

This study aims to evaluate the effectiveness of AI-assisted CXR interpretation in improving diagnostic accuracy, clinical efficiency, and patient outcomes within Brazil’s Unified Health System (SUS). Using a stepped-wedge cluster-randomized design, this protocol aims to investigate whether AI-based decision-support tools enhance general practitioners’ ability to detect predefined radiographic findings in adults presenting with respiratory symptoms or with pulmonary disease compared with unaided interpretation. The trial further seeks to determine AI’s potential to strengthen diagnostic performance in resource-limited settings and inform the implementation of scalable, equitable, and efficient health care.

## Methods

### Algorithm Development

#### Overview

Between 2019 and 2024, 3 independent deep-learning models were developed and internally validated [22]. The “Lung Abnormality” model classified CXRs as either normal or abnormal. The “Radiological Findings” model categorized patterns into predefined classes, including consolidation, pulmonary opacity, pneumothorax, atelectasis, pleural effusion, and cardiomegaly. Finally, the “Tuberculosis” model predicted whether a CXR was suggestive or not suggestive of pulmonary TB. The models were trained on CXRs from adults aged 18 years or older, including both male and female patients. The dataset comprised anteroposterior or posteroanterior views sourced from public databases and from multiple partner health care centers across Brazil, including referral clinics for TB care.

**Figure 1.**
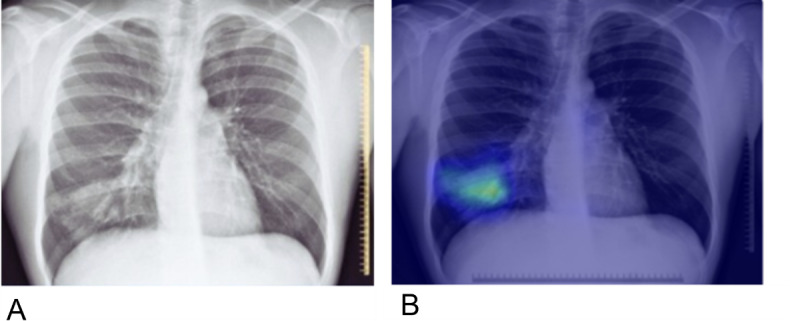
Chest X-ray (CXR) processed by the radiological findings model. (**A**) Original CXR image showing consolidation in the right lower lung field. (**B**) Artificial intelligence–generated output with heatmap highlighting the region with the highest predicted probability of consolidation.

Model predictions were given as probabilities ranging from 0 to 1, with a threshold of 0.5: values of 0.5 or higher were classified as positive, and values below 0.5 as negative. The exceptions are pneumothorax (0.8), consolidation (0.6), and TB (0.4), which had different thresholds determined by the GHOST (generalized threshold shifting) method [23]. Heatmaps highlight regions of interest, contributing to the algorithm’s output (Figure 1).

#### Internal Algorithm Validation

Internal validation proved robust performance, with the 3 models showing consistent outcomes across the validation datasets (Table 1). Overall metrics for the models were as follows: the “Pulmonary Abnormality” model achieved an area under the receiver operating characteristic curve (AUC) of 0.82, with an accuracy of 0.82, a sensitivity of 0.82, and a specificity of 0.84. The “Radiological Findings” model reached an overall AUC of 0.82, with per-class performance ranging from 0.77 (atelectasis) to 0.90 (pneumothorax). The “Tuberculosis” model achieved an AUC of 0.81, with an accuracy of 0.81, a sensitivity of 0.83, and a specificity of 0.78. These findings suggest that the models could be suitable for eventual clinical use, though additional testing in prospective real-world clinical settings is still necessary.

**Table 1.** Internal validation dataset values.

Model	AUC^a^	Sensitivity	Specificity	Accuracy
Pulmonary abnormality	0.82	0.82	0.84	0.82
Tuberculosis	0.81	0.83	0.78	0.81
Radiological findings				
Consolidation	0.78	0.84	0.57	0.59
Lung opacity	0.78	0.89	0.50	0.64
Atelectasis	0.77	0.88	0.49	0.57
Cardiomegaly	0.84	0.86	0.67	0.70
Pneumothorax	0.90	0.81	0.85	0.83
Pleural effusion	0.90	0.92	0.70	0.78

a AUC: area under the receiver operating characteristic curve.

#### External Algorithm Validation

An external retrospective validation of 1092 anonymized CXRs, including 586 double-read by thoracic radiologists at Hospital Israelita Albert Einstein, demonstrated variability in model performance. The Pulmonary Abnormality and Tuberculosis models achieved AUC values of 0.89 and 0.84, respectively, whereas the Radiological Findings model showed AUC values ranging from 0.811 to 0.96, as shown in Table 2.

**Table 2.** External validation dataset values.

Model	AUC^a^	Sensitivity	Specificity	Accuracy
Pulmonary abnormality	0.89	0.82	0.84	0.82
Tuberculosis	0.84	0.83	0.71	0.77
Radiological findings				
Consolidation	0.92	0.95	0.70	0.74
Lung opacity	0.91	0.90	0.72	0.80
Atelectasis	0.81	0.55	0.87	0.81
Cardiomegaly	0.95	0.84	0.90	0.90
Pneumothorax	0.96	1.0	0.79	0.80
Pleural effusion	0.95	0.80	0.92	0.90

a AUC: area under the receiver operating characteristic curve.

In the prospective phase of the Radiological Findings model, which included 30 real-time cases, no statistically significant differences were observed. All physicians were provided with standardized CARPL (Clinical AI Research Platform for Labeling) training to promote uniformity in image labeling [24].

Therefore, the current gap between internal and external performance per class of the Radiological Findings model underscores the need for multi-institutional datasets and rigorous real-world testing before clinical adoption. The AI models will be locked for the duration of the trial; no updates to weights or thresholds will be implemented once recruitment starts. Overall, this study underscores both the potential and limitations of AI in radiology, reinforcing that limited real-world evidence warrants caution and that robust validation frameworks are as essential as algorithm development [25-27].

### Study Design: Stepped-Wedge Randomized Design

The stepped-wedge cluster-randomized design was chosen for this trial because of its clear methodological benefits. Unlike parallel designs, all clusters eventually receive intervention, enhancing acceptability in clinical settings where prior evidence suggests potential benefit [28,29]. The phased implementation also mirrors real-world logistical constraints while allowing for both within- and between-cluster comparisons, thereby improving statistical efficiency and power [28,30]. Additionally, repeated measurements over time enable adjustment for secular trends, strengthening causal inference [28-31]. These characteristics make the stepped-wedge design especially suitable for pragmatic evaluations of health care interventions.

This nationwide, multicenter, stepped-wedge cluster-randomized trial (SW-CRT) follows the CONSORT-AI (Consolidated Standards of Reporting Trials-Artificial Intelligence) and SPIRIT-AI (Standard Protocol Items: Recommendations for Interventional Trials-Artificial Intelligence) guidelines [32,33]. It will evaluate whether AI can improve CXR interpretation in routine clinical care across Brazil. Cities will act as clusters, comprising public, private, and philanthropic health care units to ensure broad socioeconomic and geographic representation [28,30,31,34]. All clusters will begin in the control condition (standard physician interpretation) and will cross over to the intervention (AI-assisted physician interpretation) at randomly assigned monthly intervals. Potential contamination across clusters will be minimized by selecting independent clinical units with nonoverlapping staff and workflows.

**Figure 2.**
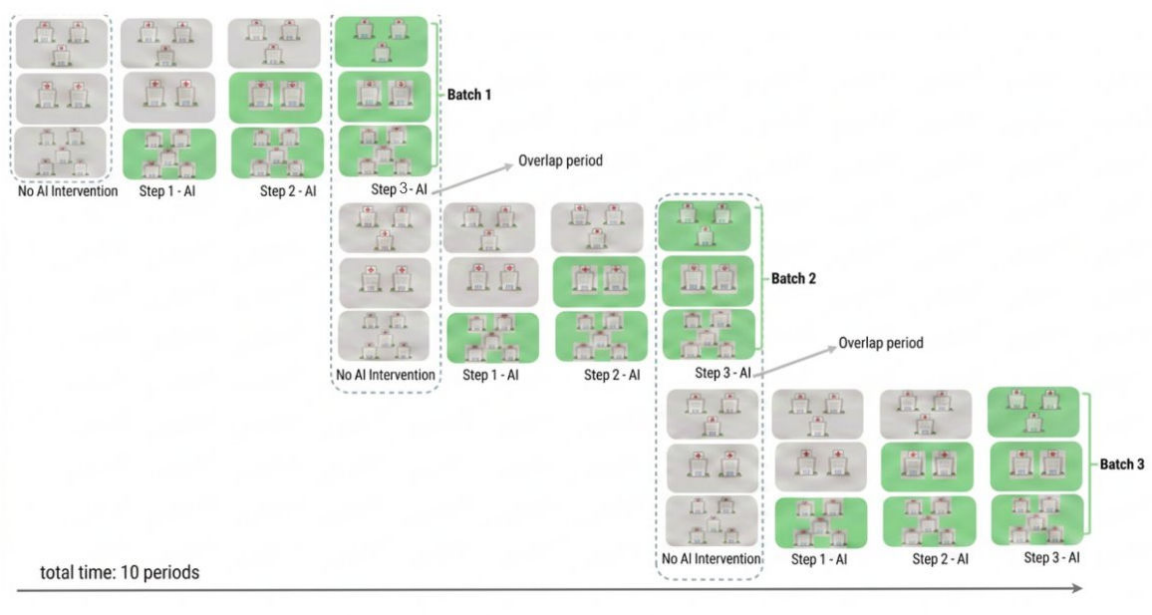
Study design and implementation flow of the LuAna (Lung Analysis) trial. The LuAna trial uses a batched stepped-wedge cluster-randomized design, where all centers begin in the control phase (gray) with physician-only interpretation. At randomly assigned sequences, clusters are grouped into batches and sequentially cross over to the intervention phase (green). Once a cluster transitions, it remains in the intervention phase until trial completion. At the site level, patient inclusion and physician interpretation occur continuously. During control periods, only the physician’s interpretation is recorded. During intervention periods, physicians first provide their interpretation, after which AI support (LuAna) is introduced. This design ensures staggered, overlapping transitions across centers while maintaining consistent trial procedures. AI: artificial intelligence.

A batched stepped-wedge approach allows staggered initiation, accommodating operational constraints while minimizing contamination or learning effects. Once a cluster transitions, it remains in the intervention arm (Figure 2). Simple urn-based randomization without replacement will occur independently in each batch. Participating centers within cities must have infrastructure for CXR interpretation (negatoscope or digital screen), reliable internet access, and commitment to study protocols.

All participating clusters will undergo a site initiation visit (SIV) prior to enrollment. All physician participants will complete the informed consent process and sign the informed consent form. During the SIV, local investigators will receive standardized training on study procedures, data entry, image capture protocols, and use of the study platform (LuAna). Training will include hands-on instruction to ensure the uniform application of inclusion criteria, proper anonymization of patient data, and completion of interpretation forms. Following the SIV, sites will begin patient recruitment and data collection. This study was registered on ClinicalTrials.gov (NCT06686251).

### Radiograph Eligibility Criteria

#### Inclusion Criteria

Eligible chest radiographs must meet all of the following criteria: it should (1) be of individuals aged 18 years or older; (2) be obtained either during the presence of respiratory symptoms or while the patient is undergoing treatment for a respiratory disease; (3) be any radiograph without a preexisting report at the time of inclusion; (4) be acquired on any X-ray equipment, provided that at least 1 frontal chest view is included; and (5) be performed either during the acute episode of the respiratory condition or throughout the postacute follow-up period, until its completion.

#### Exclusion Criteria

CXRs performed for peripherally inserted central catheter line positioning, preoperative risk assessment, lung cancer screening, or trauma-related evaluation will be excluded. In addition, radiographs printed on plain paper and examinations with technical quality below the minimum acceptable standard will also be excluded.

### Physicians

Radiograph interpretation will be performed by general practitioners or other nonradiologist physicians employed or otherwise engaged at participating trial sites. No restrictions will be imposed regarding age, sex, years since graduation, or previous training in imaging interpretation. All participating physicians will receive training using the study platform and must provide informed consent before contributing to interpretations. The training procedure emphasizes that AI outputs are advisory only; clinical responsibility remains fully with the interpreting physician, and AI suggestions never override clinical judgment. Afterward, they will complete a brief questionnaire assessing their baseline confidence in CXR interpretation, perceived challenges in reading CXRs, and initial impressions regarding the usability of the study platform (Multimedia Appendix 1).

### The LuAna Platform

LuAna is a cloud-based AI platform for CXR interpretation developed in partnership with the Brazilian Ministry of Health. The platform receives anonymized CXR with the accompanying physician report and generates 3 deep-learning outputs: (1) classification of images as normal or abnormal, (2) classification of specific radiologic findings (consolidation or pulmonary opacity, pneumothorax, atelectasis, pleural effusion, and cardiomegaly), and (3) probability scores for radiographic features suggestive of pulmonary TB. Operating fully in the cloud, LuAna provides decision support without requiring modifications to current imaging systems or clinical workflows.

### Data Collection

**Figure 3.**
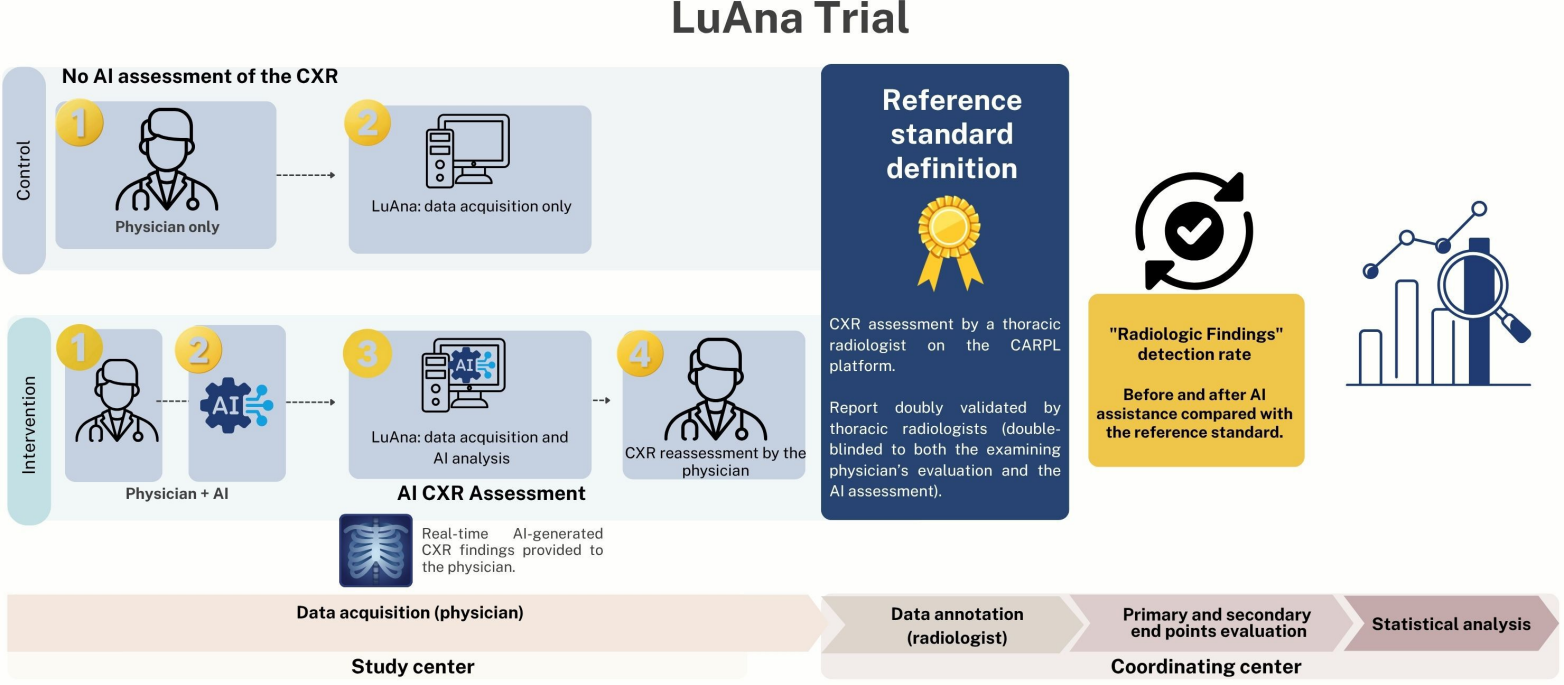
Workflow for chest X-ray (CXR) interpretation in control and intervention groups. Top panel (control group—without AI): CXRs from patients with respiratory complaints are interpreted solely by the physician (1), without access to AI support (2). Bottom panel (intervention group—with AI): physicians first perform an initial assessment of the CXR (1) and register their findings via the LuAna mobile app (2). After this, the AI model processes the image and returns a score and a heatmap highlighting abnormal regions (3). The physician can then review and, if needed, revise their interpretation based on the AI output (4). Both pre- and post-AI interpretations are recorded for analysis. For both groups, CXR assessments are compared against a reference standard, defined as thoracic radiologist evaluation performed on the CARPL, with reports independently double-validated by thoracic radiologists blinded to both the physician assessment and AI output. The primary outcome is the comparison of radiologic findings detection rates before and after AI assistance relative to the reference standard. The workflow includes data acquisition at the study centers, followed by radiologist annotation, primary and secondary end point evaluation, and statistical analysis at the coordinating center. AI: artificial intelligence; CARPL: Clinical Artificial Intelligence Research Platform for Labeling.

Anonymized data will be gathered using the LuAna platform (accessible on desktop or mobile), which records CXRs and physician interpretations (Figure 3). Physicians will access the platform using secure login credentials. All data will be automatically logged to enable auditing, reproducibility, and safety monitoring.

Only demographic variables, including age group, sex, and race, will be collected in aggregated form to characterize the study population and allow for fairness analyses, ensuring equitable and unbiased AI performance. CXRs will be captured either by photographing printed films positioned on a lit negatoscope, by photographing digital images displayed on a picture archiving and communication system monitor, or by direct screen capture, all following strict protocols to guarantee full anonymization. Physicians will capture only the thoracic image, ensuring that all patient identifiers are systematically removed and never visible. A patient ID entered solely to return the radiologist’s report to the local health unit will be encrypted and remain inaccessible to the research team. Nondiagnostic images or studies failing predefined quality-control criteria will be excluded from analysis.

Physicians will complete a brief questionnaire of their first CXR interpretation based on the following predefined questions: (1) Is the CXR normal or abnormal? (2) Are the specified radiological findings present or absent? and (3) Does the CXR show features suggestive of pulmonary TB? In the intervention group, after the physician’s clinical management, AI output (including probability scores and heatmaps) will be made available. Physicians will then document their final interpretation and decision-making process and subsequently record any changes made after reviewing the AI results. All data will be securely stored in encrypted, deidentified cloud-based repositories.

### Reference Standard Generation, Report Return, and Critical Findings

The radiologists are responsible for producing the ground-truth (reference standard) for all CXR images during the study. The first step in ground-truth generation is to assign 2 thoracic radiologists to independently interpret the CXR images to set up the reference standard, classifying each finding as “normal” or “abnormal.” The correct classification matches the reference standard. For cases classified as *abnormal*, the presence or absence of the relevant radiological findings (consolidation, pulmonary opacity, pneumothorax, atelectasis, pleural effusion, and cardiomegaly) will be assessed, as well as the presence or absence of changes suggestive of TB. Based on these evaluations by the 2 thoracic radiologists, the ground truth is established. The radiologists will be blinded to both the physicians’ interpretations and the AI outputs.

A chest radiograph will be labeled as suggestive of pulmonary TB if at least 1 radiographic feature considered compatible with pulmonary TB is present, as determined by the adjudicated thoracic radiologist reference standard. Examples include cavitation, upper-lobe–predominant consolidation or opacities, fibronodular changes or scarring with upper lobe volume loss, or a miliary pattern. If none of these features is present, the examination will be labeled as “not suggestive of pulmonary TB.”

For physician performance analyses, an examination will be considered correctly classified when the physician’s binary classification (normal vs abnormal; suggestive vs not suggestive of pulmonary TB; presence or absence of specific radiographic patterns) matches the adjudicated thoracic radiologist reference standard. In cases of any disagreement between the 2 experts in any of the evaluated items, a third radiologist, more senior, will adjudicate.

**Figure 4.**
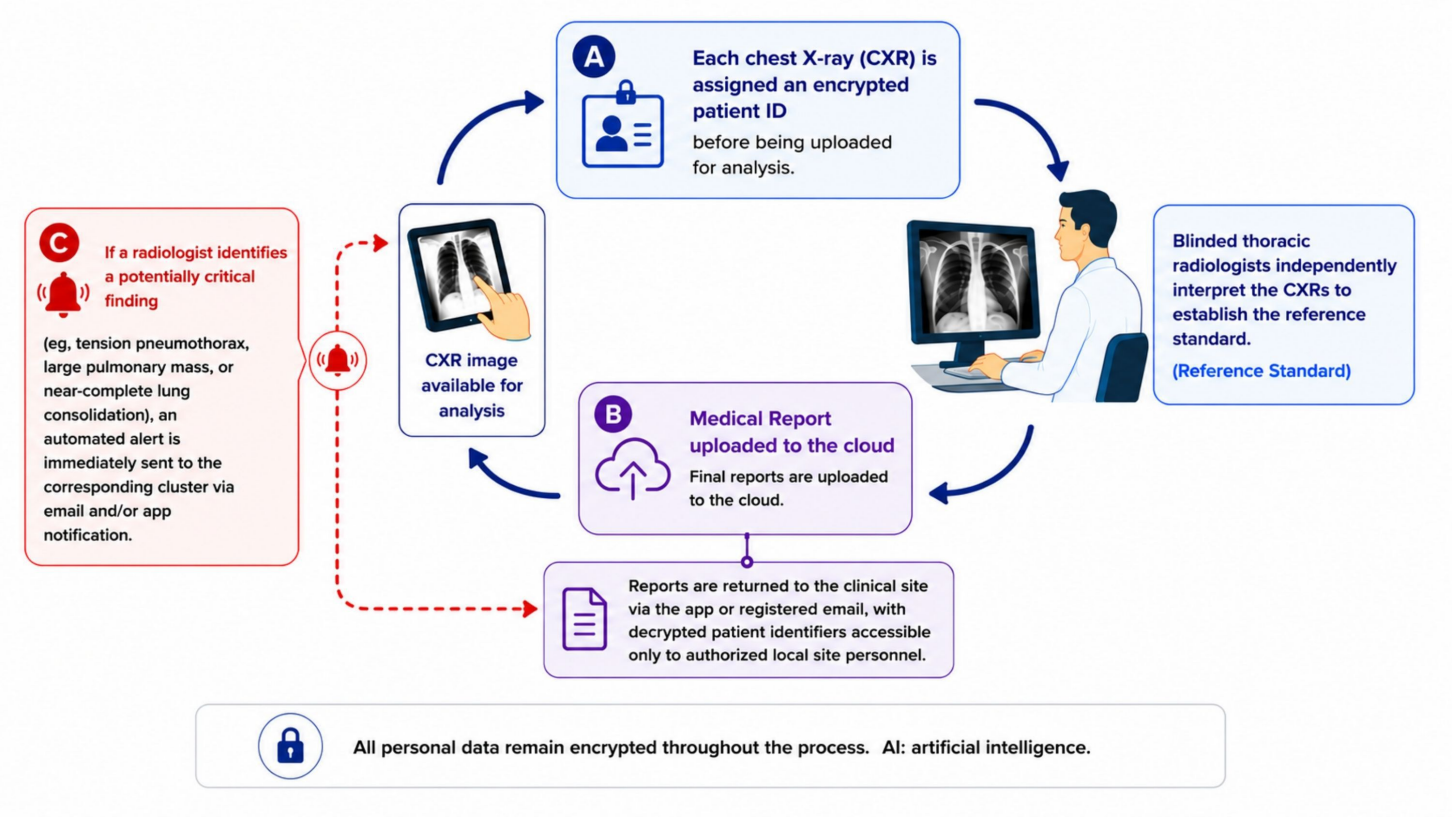
Workflow for reference standard generation, report return, and critical findings alerts. (A) Each chest X-ray (CXR) is assigned an encrypted patient ID before being uploaded for analysis. (B) Blinded thoracic radiologists independently interpret the images to establish the reference standard. Final reports are uploaded to the cloud and returned to the clinical site via the app or registered email, with decrypted patient identifiers accessible only to the site. (C) If a radiologist identifies a potentially critical finding, such as tension pneumothorax, a large pulmonary mass, or near-complete lung consolidation, an automated alert is immediately sent to the cluster via email or app notification. All personal data remain encrypted throughout the process. AI: artificial intelligence.

All findings will be registered in the CARPL, with abnormalities annotated to allow direct comparison with AI outputs. This will serve as the reference value for model validation and performance analysis (Figure 4).

Patient identifiers will be used solely to return reports of critical imaging findings to clinical sites, when present. These identifiers are cryptographically isolated from the research dataset and are never accessible to the research team (Figure 4). If the radiologist identifies a potentially critical finding, such as tension pneumothorax, a large obstructive mass, or near-complete lung consolidation, an alert will be sent to the originating site via email and/or phone (Figure 4). Patient data will remain encrypted; only the automated system will link the alert to the corresponding patients, ensuring privacy and prompt clinical follow-up. An institutional lifetime license ensures consistent labeling with the same software version throughout the study.

At the end of the study, all participating physicians and radiologists will be invited to complete a standardized questionnaire assessing the usability of the LuAna platform and its integration into routine clinical workflows. Responses will be used to address implementation challenges and opportunities for improvement (Multimedia Appendix 1).

### Data and Safety Monitoring Board

An independent Data and Safety Monitoring Board, comprising external experts in clinical trials, radiology, statistics, and ethics with no conflicts of interest, will oversee the study in accordance with international guidelines and good clinical practice. The board will be responsible for safeguarding patient safety and ensuring study integrity.

### Statistical Analysis

The sample size was calculated to detect a minimum difference of 10% in the primary outcome—a binary indicator of whether the physician correctly labeled a CXR—between the AI-assisted and control groups. The calculation accounted for the stepped-wedge cluster-randomized design with batched implementation using the following assumptions: 9 clusters randomized in 3 batches to cross over from control to intervention, 10 time periods, a baseline detection rate of 67% in the control group, an expected increase to 77% in the intervention group, and an intracluster correlation coefficient ranging from 0.001 to 0.003.

The design effect was adjusted for the correlation structure induced by repeated measures within clusters over time using methods described by Hussey and Hughes [28] and extended to account for batched randomization, as described by Hemming et al [35,36]. The power calculation targeted 80% power at a 2-sided significance level of 5% and accounted for potential variability in cluster sizes. The final average cluster size was 37, and the overall sample size was inflated by 10% to account for anticipated exclusions due to image quality issues or missing data (dropout). This approach ensures adequate power to detect a meaningful effect of the intervention while maintaining the integrity of the stepped-wedge design with staggered implementation across batches.

Primary analysis will follow the modified intention-to-treat principle. All eligible CXRs (the unit of analysis in this study) will be analyzed according to the study group (control or intervention), to which their corresponding cluster was allocated at the time of image acquisition, regardless of physician adherence to the intervention or exposure to AI output. Images of poor quality, as defined by our radiologists, will be excluded postrandomization.

The primary outcome analysis will use a generalized linear mixed model with a binomial distribution and logit link function to estimate the effect of the AI-assisted intervention on detection probability. The model will include fixed effects for the intervention group (AI-assisted vs control), calendar time (to adjust for secular trends), time since intervention rollout (to account for learning or adaptation effects), and batch effects. In addition, random intercepts for clusters will be specified to account for intracluster correlation.

Results will be reported as adjusted odds ratios with 95% CIs and 2-sided *P* values. Model fit and assumptions will be evaluated using the Residual Diagnostics for Hierarchical (Multilevel/Mixed) Regression Models (DHARMa) [37]. In case of convergence issues or model instability, generalized estimating equations with robust SEs will be used as an alternative to estimate population-averaged effects. Independent models will also be fitted for each radiological finding, as well as for the pulmonary abnormality and TB algorithm.

Model performance metrics will be reported with 95% CIs [38,39]. Responses to questions regarding the mobile interface will be summarized as frequencies. Chi-square test and ordinal logistical regression will be used to investigate associations between these responses and physician characteristics (eg, years in practice and biological sex). Additionally, generalized linear mixed models will be fitted with the algorithm’s output as the dependent variable, adjusted for image characteristics, to investigate their effect on AI classification.

Missing data are expected to be minimal due to app-enforced completeness checks. Incomplete records will be described, and sensitivity analyses using multiple imputation will be performed if the proportion of missing data exceeds 10%. All analyses will be performed in R (R Foundation for Statistical Computing), and code scripts will be version-controlled and archived to ensure reproducibility.

### Ethical Considerations

This study was reviewed and approved by the local Ethics Committee of Hospital Israelita Albert Einstein (CEP Einstein), São Paulo, Brazil, registered under IRB00005041, in accordance with applicable national and international regulations (CAAE number 82494524.0.1001.0071). A waiver of written informed consent for patients was granted, as the study is strictly observational and involves no intervention in routine clinical care, no modification of clinical management, and no access to patients’ medical records. Only chest radiographs obtained as part of standard care were used, and all images were fully anonymized prior to storage and analysis, with no directly identifiable information included in the research dataset. However, written informed consent was obtained from the physicians responsible for enrolling patients in the LuAna trial, as they are considered direct research participants.

All data are handled under strict confidentiality procedures. Chest radiograph images were collected via the LuAna app and stored in an anonymized format on a secure cloud-based server.

The analytical database contains exclusively deidentified data. Access to identifiable information at the study sites, when applicable, is restricted to authorized local investigators. Study data will be securely stored at the hosting institution for a minimum of 5 years, in compliance with institutional and regulatory requirements.

Posttrial access pathways for the tool will be discussed with the Brazilian Ministry of Health to ensure continuity if the intervention proves beneficial. Technical details of the algorithm, including its development, training, validation, and performance characteristics, will be described in accordance with the CLAIM (Checklist for Artificial Intelligence in Medical Imaging) reporting guidelines [40], complementing the CONSORT-AI and SPIRIT-AI standards applied to the clinical trial components [32,33].

### Outcomes

#### Primary Outcome

The primary outcome is expected to show that AI support improves physicians’ ability to detect radiographic abnormalities, including consolidation or pulmonary opacity, pneumothorax, atelectasis, pleural effusion, and cardiomegaly. Physicians are anticipated to be more likely to correctly identify at least 1 of these 5 findings when assisted by AI than when interpreting CXRs unaided. This outcome is defined as a patient-level binary indicator of whether the physician correctly identifies any of the 5 prespecified findings compared with the thoracic radiologist’s reference standard.

#### Secondary Outcomes

The secondary outcomes are expected to demonstrate that AI assistance increases detection rates for each of the 5 radiographic findings, as well as overall pulmonary abnormality and TB-related patterns, relative to unaided physician interpretation. Improvements in abnormality localization accuracy are also anticipated, both for the AI outputs alone and for physician interpretations after reviewing AI results. Diagnostic performance compared with the reference standard is expected to improve, with higher sensitivity, specificity, AUC, positive predictive value, and negative predictive value, reflecting greater reliability in identifying clinically relevant radiological abnormalities. The secondary outcomes are depicted in Textbox 1.

Textbox 1. **Secondary objectives and outcomes.**

Secondary objectives

- Measure the effect of artificial intelligence (AI) on physicians’ detection rates for each of the 5 radiologic findings independently, pulmonary abnormality, and features suggestive of pulmonary tuberculosis
- Evaluate AI algorithm performance in a real-world setting
- Evaluate physicians’ experiences regarding usability of the mobile interface
- Assess the impact of external influencing factors on AI algorithm predictions
- Evaluate physicians’ learning related to interpretation of radiologic findings on chest X-rays (CXRs) before and after the intervention

Secondary outcomes

- Whether the physician correctly identifies each of the 5 radiologic findings, pulmonary abnormality, and features suggestive of pulmonary tuberculosis on chest radiographs
- Comparison between AI algorithm outputs for CXRs and thoracic radiologist reference standard classifications to calculate sensitivity, specificity, area under the receiver operating characteristic curve, positive predictive value, and negative predictive value
- Responses to questions regarding the mobile app interface, responsiveness, and functionality measured using a 7-point Likert scale
- Associations between AI algorithm outputs for CXRs and CXR characteristics, including photo quality, presence of thoracic devices, and technical artifacts, as labeled by radiologists
- Change in physician interpretation performance over time and according to the duration of participation in the study

## Results

The LuAna trial received institutional ethics board approval and was formally activated in October 2024. Data collection began in January 2026 with a projected end date of September 2026. At interim review (November 2025), 3 of the 13 participating centers had completed all site activation requirements and were prepared to initiate enrollment. The trial is currently ongoing with active data collection. Data analysis is scheduled for completion by November 2026, and results dissemination is expected by January 2027.

## Discussion

### Principal Findings

This trial is a pivotal effort to evaluate how AI can enhance diagnostic accuracy, clinical workflows, and physician performance in interpreting chest radiographs.

By using a batched stepped-wedge cluster-randomized design across a nationally representative sample of health care settings, we aim to rigorously assess the real-world utility of LuAna, an AI-powered platform for CXR interpretation developed in the public sector to assist general practitioners.

Despite ongoing debate about the advantages of SW-CRTs, including concerns about equipoise and delayed access to interventions [41], this design offers distinct benefits for implementation research. Governed by the same ethical principles as all clinical research [42], SW-CRTs are particularly valuable when evaluating innovations that are expected to be beneficial and for which withholding access may be contentious [42]. In the SUS context, this approach ensures that all centers ultimately gain access to AI support, promotes fairness and acceptability, and provides a pragmatic pathway to sustainable integration of the intervention into routine care.

The central goal is to determine whether LuAna improves clinicians’ ability to detect radiographic abnormalities, particularly in environments with limited access to radiology expertise. Comparisons against adjudicated, double-blinded radiologist interpretations will enable the quantification of diagnostic agreement, while stratified analyses by site type and physician characteristics will offer insights into contextual factors that may influence AI performance.

Importantly, the trial also investigates the clinical impact beyond diagnostic accuracy. By evaluating patient management decisions and turnaround times, we aim to capture how AI affects treatment pathways and efficiency in routine care. Usability assessments will provide critical feedback for refining and scaling the platform, thereby ensuring seamless integration into diverse clinical workflows across both mobile and desktop interfaces.

Robustness will be evaluated through sensitivity analyses on image quality, artifacts, and medical devices, thereby validating the model’s generalizability under real-world conditions. Additionally, longitudinal analysis of physician interpretations will explore whether repeated AI exposure improves independent performance, highlighting AI’s potential educational role.

Beyond its methodological rigor, the LuAna trial advances an area in which robust evidence remains scarce: the real-world, prospective clinical evaluation of AI tools. While algorithmic performance is well documented in retrospective studies, few investigations have examined how AI behaves once deployed within the operational complexity of large, heterogeneous public health systems [40,43,44]. This gap has been repeatedly highlighted by international frameworks, including the DECIDE-AI (Developmental and Exploratory Clinical Investigation of Decision Support Systems Driven-Artificial Intelligence) guidelines, which emphasize the need for pragmatic, early-stage clinical evaluation before widescale deployment. Indeed, studies show positive primary outcomes for AI in clinical practice, but concerns remain due to mostly single-center trials, limited demographic data, and inconsistent reports on operational efficiency, which affect generalizability and practical use [45,46].

Evaluating AI-based decision-support systems involves several challenges: understanding their nature as complex interventions; considering user variability and the biases it introduces; integrating human factors into the collaboration between clinicians and AI; assessing both real patients and their data; dealing with continuous system changes; preventing the reproduction of health inequalities; ensuring that results generalize to different contexts; and guaranteeing reproducibility in the real world. By implementing LuAna across a nationally diverse range of health care settings, this study not only provides valuable empirical data on AI performance in real-world situations but also offers a unique opportunity to examine issues related to fairness [47-49].

Variability in patient demographics, imaging equipment, staffing, and workflow conditions across Brazil’s public health system creates a natural stress test for equity: the trial can assess whether AI-assisted interpretation benefits all subpopulations and settings equally or whether performance disparities emerge [50,51]. In doing so, it addresses 2 critical gaps in the global AI literature—real-world clinical validation and algorithmic fairness—offering evidence that is seldom available yet essential for responsible, scalable, and equitable integration of AI into routine care.

Grounded in operational realities and health care equity, this study contributes to a broader movement toward responsible, evidence-based AI integration in clinical practice. It aligns with international recommendations for validation frameworks and addresses a global need for tools that are both technically robust and contextually appropriate for low- and middle-income health systems.

### Limitations

Although the stepped-wedge design enhances real-world applicability by including diverse health care settings, the findings may still be influenced by context-specific factors, such as infrastructure, physician training, and patient demographics. Variation in image acquisition, whether from printed films, computer displays, or mobile capture, may introduce heterogeneity that affects both physician interpretation and algorithm performance. In addition, while the trial evaluates diagnostic accuracy, turnaround times, usability, and model robustness, it does not directly measure patient-level outcomes such as morbidity or mortality. Equity analyses are further limited by the narrow range of demographic variables collected, which may not fully capture socioeconomic or clinical determinants of performance. Finally, the lack of detailed clinical data, including comorbidities, treatment history, and laboratory findings, restricts the ability to contextualize radiographic interpretations and fully assess the AI integration into patient care pathways.

### Conclusion

Future work should prioritize expanding datasets to include a broader spectrum of acquisition standards, additional radiographic projections (eg, lateral views), and complementary imaging modalities such as CT. Scaling to additional centers across Brazil—capturing greater geographic, demographic, and service-level diversity—will further strengthen dataset representativeness and enhance algorithm robustness. The adoption of standardized annotation protocols will support higher-quality data curation and reduce uncertainty in the reference standard. In parallel, strategies such as transfer learning and active learning should be explored to optimize performance in data-limited environments.

Ultimately, LuAna seeks to demonstrate that AI-powered diagnostic support can be safely and effectively integrated into public health care, empowering clinicians and improving outcomes for underserved populations. This study will lay the groundwork for broader AI adoption in medical imaging, grounded in evidence, equity, and clinical impact.

If proven effective, LuAna could help bridge diagnostic gaps by supporting nonspecialist clinicians, accelerating decision-making, and strengthening health care delivery in underserved regions, offering a scalable, evidence-based model for equitable AI integration in medical imaging.

## Supplementary material

10.2196/88626 Multimedia Appendix 1 Application evaluation questionnnaire for physicians

10.2196/88626 Checklist 1 SPIRIT Checklist

## Abbreviations

AI: artificial intelligence
AUC: area under the receiver operating characteristic curve
CARPL: Collaborative Artificial Intelligence Annotation Platform
CLAIM: Checklist for Artificial Intelligence in Medical Imaging
CONSORT-AI: Consolidated Standards of Reporting Trials-Artificial Intelligence
CT: computed tomography
CXR: chest X-ray
DECIDE-AI: Developmental and Exploratory Clinical Investigation of Decision Support Systems Driven-Artificial Intelligence
DHARMa: Residual Diagnostics for Hierarchical (Multilevel/Mixed) Regression Models
GHOST: generalized threshold shifting
LMICs: low- and middle-income countries
LuAna: Lung Analysis
SIV: site initiation visit
SPIRIT-AI: Standard Protocol Items: Recommendations for Interventional Trials-Artificial Intelligence
SUS: Sistema Único de Saúde
SW-CRT: stepped-wedge cluster-randomized trial
TB: tuberculosis
