# Supplementary material for: AI-Assisted Chest X-Ray Interpretation in Resource-Limited Settings: LuAna Stepped-Wedge Trial Protocol
Source: JMIR Res Protoc. 2026 Jul 13;15:e88626. doi: 10.2196/88626 (PMC13361621; doi:10.2196/88626)
Supplement: Multimedia Appendix 1 [file resprot-v15-e88626-s001.pdf]

Supplementary Material

Application Evaluation Questionnaire for Physicians

---

1. What is your level of confidence in interpreting a chest X-ray?

1. I feel very confident interpreting a chest X-ray.
2. I feel confident interpreting a chest X-ray.
3. I feel somewhat confident interpreting a chest X-ray.
4. I do not feel confident interpreting a chest X-ray.
5. I feel no confidence at all in interpreting a chest X-ray.

---

2. In your opinion, what is the greatest difficulty in interpreting a chest X-ray?

1. Identifying the lesion(s) in the image.
2. Correlating the image with clinical and laboratory data.
3. Differentiating a normal radiograph from an abnormal one.
4. Dealing with technical challenges inherent to the exam (e.g., poor patient positioning).
5. Developing diagnostic hypotheses.
6. Other (add a field to specify).

Usability – To what extent do you agree or disagree with each of the following statements, where 1 means “Strongly disagree” and 7 means “Strongly agree”?

**Statement**	1	2	3	4	5	6	7
The LuAna app is easy to use.	o	o	o	o	o	o	o
Navigation in the app is consistent when moving between screens.	o	o	o	o	o	o	o
The app interface enabled me to utilize all the functions (such as entering information, uploading images, and viewing information) offered by the app.	o	o	o	o	o	o	o
I found the information in the app to be clear and easy to interpret.	o	o	o	o	o	o	o
The app is useful for my medical practice.	o	o	o	o	o	o	o

Satisfaction and Acceptance

To what extent do you agree or disagree with each of the following statements, with 1 being “Strongly Disagree” and 7 being “Strongly Agree”?

Statement	1	2	3	4	5	6	7
I am satisfied with the application's design.	○	○	○	○	○	○	○
I am satisfied with the quality and clarity of the information provided by the application.	○	○	○	○	○	○	○
I am satisfied with the application as a tool to support the diagnosis of radiological findings.	○	○	○	○	○	○	○
I want to continue using the LuAna application.	○	○	○	○	○	○	○
I would recommend the application to other physicians.	○	○	○	○	○	○	○

Effectiveness Questionnaire

To what extent do you agree or disagree with each of the following statements, with 1 being "Totally disagree" and 7 being "Totally agree"?

	1	2	3	4	5	6	7
The application made me consider changing my diagnostic hypothesis.	o	o	o	o	o	o	o
The application made me consider changing my medical conduct.	o	o	o	o	o	o	o
The application made me feel more confident in defining radiological findings on chest X-rays	o	o	o	o	o	o	o
I consider the application's suggestions of radiological findings to be reliable.	o	o	o	o	o	o	o
The application helped me conduct a faster and more dynamic consultation.	o	o	o	o	o	o	o
