# Supplementary material for: AI-Assisted Chest X-Ray Interpretation in Resource-Limited Settings: LuAna Stepped-Wedge Trial Protocol
Source: JMIR Res Protoc. 2026 Jul 13;15:e88626. doi: 10.2196/88626 (PMC13361621; doi:10.2196/88626)
Supplement: Checklist 1 [file resprot-v15-e88626-s002.docx]

**SPIRIT-AI Checklist**

| **Section** | **Item** | **SPIRIT 2013 Item** | **SPIRIT-AI Extension** | **Manuscript page(s)** |
| --- | --- | --- | --- | --- |
| Administrative Information | | | | |
| **Title** | 1 | Descriptive title identifying the study design, population, interventions, and, if applicable, trial acronym | SPIRIT-AI 1 (i) Elaboration: Indicate that the intervention involves artificial intelligence/machine learning and specify the type of model. | 1 |
|  |  |  | SPIRIT-AI 1 (ii) Elaboration: Specify the intended use of the AI intervention. | 1; 3; 5 |
| **Trial registration** | 2a | Trial identifier and registry name. If not yet registered, name of intended registry. |  | 6 |
|  | 2b | All items from the World Health Organization Trial Registration Dataset |  | 5 |
| **Protocol version** | 3 | Date and version identifier |  | 6 |
| **Funding** | 4 | Sources and types of financial, material, and other support Role of study sponsor and funders in study design, conduct, analysis, interpretation, and publication decisions |  | 12 |
| **Roles and responsibilities** | 5a | Names, affiliations, and roles of protocol contributors |  | 1; 12 |
|  | 5b | Name and contact information for the trial sponsor |  | 12 |
|  | 5c | Role of study sponsor and funders in study design, conduct, analysis, interpretation, and publication decisions |  | 12 |
|  | 5d | Composition and responsibilities of coordinating center, steering committee, endpoint adjudication committee, data management team, etc. |  | 7; 8; 12 |
| Introduction | | | | |
| **Background and rationale** | 6a | Description of research question and justification, including summary of relevant studies | SPIRIT-AI 6a (i) Extension: Explain intended use of AI within clinical pathway and intended users.  SPIRIT-AI 6a (ii) Extension: Describe any pre-existing evidence for the AI intervention. | 2; 3; 5 |
|  | 6b | Explanation for choice of comparators |  | 6-8; 10 |
| **Objectives** | 7 | Specific objectives or hypotheses |  | 3; 5 |
| **Trial design** | 8 | Description of trial design, allocation ratio, and framework |  | 5-8 |
| Methods: Participants, Interventions and Outcomes | | | | |
| **Study setting** | 9 | Description of study settings and countries | SPIRIT-AI 9 Extension: Describe onsite and offsite requirements to integrate AI into trial setting. | 5 |
| **Eligibility criteria** | 10 | Inclusion and exclusion criteria | SPIRIT-AI 10 (i) Elaboration: Criteria at participant level.  SPIRIT-AI 10 (ii) Extension: Criteria at input data level. | 6 |
| **Interventions** | 11b | Criteria for discontinuing or modifying interventions |  | N/A |
|  | 11c | Strategies to improve adherence and monitoring |  | 6 |
|  | 11d | Permitted and prohibited concomitant care |  | N/A |
| **Outcomes** | 12 | Primary, secondary and other outcomes, metrics, aggregation and timepoints |  | 10 |
| **Participant timeline** | 13 | Schedule of enrollment, interventions, assessments |  | 5; 6 |
| **Sample size** | 14 | Estimated number and statistical assumptions |  | 9 |
| **Recruitment** | 15 | Strategies for achieving enrollment |  | 6; 10 |
| **Sequence generation** | 16a | Method of allocation sequence generation |  | 5 |
| **Allocation concealment** | 16b | Mechanism of allocation concealment |  |  |
| **Implementation** | 16c | Who generates sequence, enrolls, assigns |  |  |
| **Blinding** | 17a | Who will be blinded and how |  | 7; 8 |
|  | 17b | Conditions and procedure for unblinding |  | N/A |
| Methods: Data Collection, Management and Analysis | | | | |
| **Data collection methods** | 18a | Plans for assessment and collection of data |  | 7; 8 |
|  | 18b | Plans to promote retention and complete follow-up |  | N/A |
| **Data management** | 19 | Plans for data entry, coding, security and storage |  | 7-9 |
| **Statistical methods** | 20a | Statistical methods for primary and secondary outcomes |  | 9 |
|  | 20b | Methods for additional analyses |  | 9 |
|  | 20c | Definition of analysis population and handling of missing data |  | 9 |
| Methods: Monitoring | | | | |
| **Data monitoring** | 21a | Composition and independence of DMC |  | 8;9 |
|  | 21b | Interim analyses and stopping guidelines |  | N/A |
| **Harms** | 22 | Plans for adverse events reporting | SPIRIT-AI 22 Extension: Specify plans to identify and analyze AI performance errors. | 8; 9 |
| **Auditing** | 23 | Frequency and independence of auditing |  | N/A |
| **Ethics and Dissemination** |  | | | |
| **Research ethics approval** | 24 | Plans for REC/IRB approval |  | 9 |
| **Protocol amendments** | 25 | Communication of protocol modifications |  | N/A |
| **Consent or assent** | 26a | Who will obtain consent and how |  | 6; 9 |
|  | 26b | Additional consent provisions |  | N/A |
| **Confidentiality** | 27 | Protection of participant data |  | 7-9 |
| **Declaration of interests** | 28 | Competing interests |  | 12 |
| **Access to data** | 29 | Access to final dataset | SPIRIT-AI 29 Extension: State whether and how AI intervention and/or code can be accessed. | 12 |
| **Ancillary and post-trial care** | 30 | Post-trial care and compensation |  | N/A |
| **Dissemination policy** | 31a | Communication of trial results |  | 12 |
|  | 31b | Authorship eligibility guidelines |  | 12 |
|  | 31c | Public access to protocol, dataset, and statistical code |  | N/A |
| Appendices | | | | |
| **Informed consent materials** | 32 | Model consent form and related documentation |  | 6;9 |
| **Biological specimens** | 33 | Collection and storage of biological specimens |  | N/A |
